# Seed Treatment with *Trichoderma longibrachiatum* T6 Promotes Wheat Seedling Growth under NaCl Stress Through Activating the Enzymatic and Nonenzymatic Antioxidant Defense Systems

**DOI:** 10.3390/ijms20153729

**Published:** 2019-07-30

**Authors:** Shuwu Zhang, Bingliang Xu, Yantai Gan

**Affiliations:** 1 College of Plant protection, Gansu Agricultural University, Biocontrol Engineering Laboratory of Crop Diseases and Pests of Gansu Province, Lanzhou 730070, China; 2 Agriculture and Agri-Food Canada, Government of Canada Swift Current Research & Development Centre, Swift Current, SK S9H 3X2, Canada

**Keywords:** *Trichoderma* spp., different levels of salt stress, wheat seedling, plant growth promotion, reactive oxygen species, scavenging enzymes, gene expression

## Abstract

Salt stress is one of the major abiotic stresses limiting crop growth and productivity worldwide. Species of *Trichoderma* are widely recognized for their bio-control abilities, but little information is regarding to the ability and mechanisms of their promoting plant growth and enhancing plant tolerance to different levels of salt stress. Hence, we determined (i) the role of *Trichoderma longibrachiatum* T6 (TL-6) in promoting wheat (*Triticum aestivum* L.) seed germination and seedling growth under different levels of salt stress, and (ii) the mechanisms responsible for the enhanced tolerance of wheat to salt stress by TL-6. Wheat seeds treated with or without TL-6 were grown under different levels of salt stress in controlled environmental conditions. As such, the TL-6 treatments promoted seed germination and increased the shoot and root weights of wheat seedlings under both non-stress and salt-stress conditions. Wheat seedlings with TL-6 treatments under different levels of NaCl stress increased proline content by an average of 11%, ascorbate 15%, and glutathione 28%; and decreased the contents of malondialdehyde (MDA) by an average of 19% and hydrogen peroxide (H_2_O_2_) 13%. The TL-6 treatments induced the transcriptional level of reactive oxygen species (ROS) scavenging enzymes, leading to the increases of glutathione s-transferase (GST) by an average of 17%, glutathione peroxidase (GPX) 16%, ascorbate peroxidase (APX) 17%, glutathione reductase (GR) 18%, dehydroascorbate reductase (DHAR) 5%. Our results indicate that the beneficial strain of TL-6 effectively scavenged ROS under NaCl stress through modulating the activity of ROS scavenging enzymes, regulating the transcriptional levels of ROS scavenging enzyme gene expression, and enhancing the nonenzymatic antioxidants in wheat seedling in response to salt stress. Our present study provides a new insight into the mechanisms of TL-6 can activate the enzymatic and nonenzymatic antioxidant defense systems and enhance wheat seedling tolerance to different levels of salt stress at physiological, biochemical and molecular levels.

## 1. Introduction

Salinity stress is one of the serious abiotic stresses and global environmental problems that adversely affecting and limiting the plant growth and yield, agricultural production and environmental health worldwide [1,2]. At present time, more than 800 million hectares of soil resources worldwide have been severely damaged by soil salinity, representing 7% of the total lands surface [3,4]. As a consequence, it is predicted that more than 20% of global agricultural production was affected [5] by salinity worldwide. In order to alleviate the adverse effects caused by salt stress, many researchers have tried to breed and develop salt-tolerant plant cultivars to alleviate this situation. However, little success has been achieved in developing more efficient salt-tolerant plants [6], and also further studies are required to better understand the mechanisms’ response for the plants’ tolerance to salt stress. It is therefore worthwhile to explore the question of how to mitigate the adverse effects of salt stress, and enhance plant tolerance to salt stress, and eventually increase the plant yields.

A new and innovative strategy that has attracted an increasing attention in recent years, is to use the remarkable beneficial bacteria and fungi to induce plant resistance to abiotic stress. This approach will open a new avenue for capitalizing on the cultivable microbiome to strengthen plant tolerance to salt stress, and thus to refine agricultural practices and production under saline conditions [7]. *Trichoderma* spp. are one of the free-living fungi that are well recognized as bio-control agents of soil-borne plant pathogens commonly in soil and root ecosystems [8]. As such, many researchers have been widely studied for their capacity to produce antibiotics, parasitize other fungi and nematodes, and compete with deleterious plant microorganisms [9]. In addition, some *Trichoderma* strains were revealed that they can interact directly with roots, increase plant growth and tolerance to different abiotic stresses [10]; plants roots colonized by *T. harzianum* results in increased level of plant enzymes that helped enhance plant resistance to abiotic stresses, including various peroxidases, chitinases, β-1, 3-glucanases, lipoxygenase-pathway hydro peroxide lyase and compounds like phytoalexins and phenols [11,12,13]; *Arabidopsis* and cucumber (*Cucumis sativus* L.) roots exposed to salt stress and inoculated with *Trichoderma* spp. revealed an increased expression of genes related to salt-tolerance [14]. Although there are numerous reports on the abilities of how *Trichoderma* spp. alleviate the adverse effects of abiotic stresses or enhance plant tolerance to abiotic stresses, the specific knowledge for the mechanisms of *Trichoderma* spp. in alleviating or enhancing plant tolerance to salt stress is a very complex phenomenon and the nature remains unresolved [15].

Our previous studies revealed that *T. longibrachiatum* has a higher potential in promoting plants growth under abiotic (150 mM salt stress) and biotic (nematodes infection) stresses [16,17]. However, the previous studies failed to determine the potential of *T. longibrachiatum* T6 (TL-6) in promoting plant growth and enhancing plant tolerance to salt stress under different levels, as well as the possible physiological, biochemical and molecular mechanisms of TL-6 enhancing the tolerance of wheat to different levels of salt stress. Therefore, the aims of the present study were to (i) investigate the role of TL-6 in promoting wheat seed germination and seedling growth under different levels of salt solutions, and (ii) the possible mechanisms responsible for the enhanced tolerance of wheat to salt stress through limiting the reactive oxygen species (ROS) damage by TL-6. 

## 2. Results 

### 2.1. Effect of TL-6 on Wheat Seed Germination and Seedling Growth in Vitro 

Compared with wheat seeds that were soaked with sterile water, the seeds treated with the strain of TL-6 increased the germination rate by an average of 6%, germination potential 8%, and germination index 10%, across the three NaCl treatments (0, 10 and 20 mg mL^−1^). Without the TL-6 treatment, the germination rate, germination potential and germination index were all decreased significantly with the increase of NaCl concentration (Table 1).

**Figure 1:**
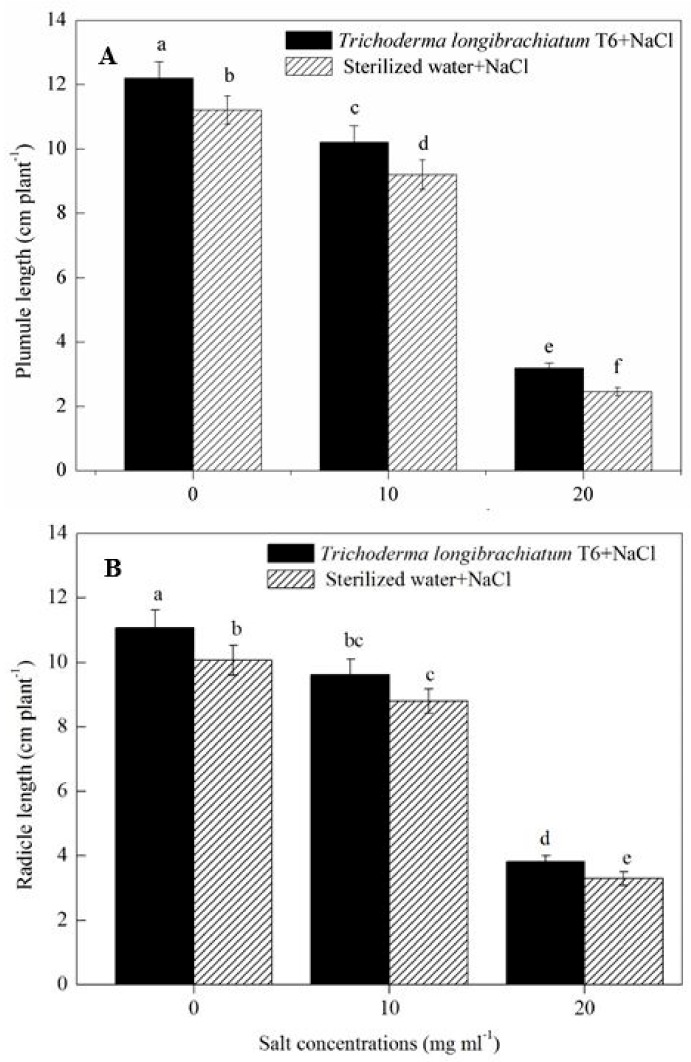
Effect of the strain *Trichoderma longibrachiatum* T6 on wheat seedling growth after treated with NaCl solutions, with (**A**) wheat seed plumule length and (**B**) radicle length. The line bars represent the standard errors of the means. Different letters denote significant difference at the *p* < 0.05 level by Duncan’s new multiple range test (n = 12). The treatments are detailed in the footnote of Table 1.

Increased NaCl concentration (from 0, 10 to 20 mg mL^−1^) significantly decreased the growth of wheat seedlings (Figure 1). At a given NaCl level, the TL-6 strain treatment increased the length of plumule and radicle of wheat seedlings significantly compared with sterile water treatment. The treatment of TL-6 strain increased the length of plumule of wheat seedlings by 9% at the 0 mg mL^−1^ of NaCl treatment, and furthered to 11% at 10 mg mL^−1^, and 30% at 20 mg mL^−1^ (Figure 1A), whereas the TL-6 treatment increased the length of radicle by 10% at 0 mg mL^−1^, 9% at 10 mg mL^−1^, and 16% at 20 mg mL^−1^ (Figure 1B).

### 2.2. Effect of TL-6 on Wheat Seedling Growth in Greenhouse

Increased concentration of NaCl from 0 to 20 mg mL^−1^ decreased both shoot height and root length of wheat seedlings significantly regardless under sterile water or TL-6 treatment (Table 2). However, the TL-6 treatment increased the shoot height and root length of wheat seedlings significantly compared with sterile water treatments whether the NaCl concentration was at 0, 10, or 20 mg mL^−1^. Averaged across the three NaCl concentration treatments, the seedlings treated with the strain of TL-6 increased the shoot height by 9% to 18% and root length by 11% to 16%; similarly, the TL-6 treatment increased the wheat seedling fresh weight by 15% to 23% and dry weight by 17% to 29% (Table 2).

### 2.3. Lipid Peroxides, H_2_O_2_ and Proline Content in Wheat Seedling

The MDA and H_2_O_2_ contents in wheat seedlings treated with sterile water were increased significantly with the increase of NaCl concentrations from 0 to 20 mg mL^−1^. Measured at Day 35, the content of MDA was increased by 36% to 41% and the H_2_O_2_ content was increased by 14% to 24% with the NaCl solution increasing from 10 to 20 mg mL^−1^, compared with 0 mg mL^−1^ of NaCl concentration under sterile water treatment (Table 3). The seedlings treated with TL-6 strain decreased MDA content by 15% at the NaCl concentration of 0 mg mL^−1^, furthered to 22% at 10 mg mL^−1^ and 19% at 20 mg mL^−1^, whereas the TL-6 treatment decreased H_2_O_2_ contents by 10%, 12% and 16%, respectively, compared with sterile water treatment. The magnitude of the decrease of MDA contents in the TL-6 treated seedlings was greater with 10 mg mL^−1^ of NaCl concentration, while the decrease of H_2_O_2_ contents was greater with 20 mg mL^−1^ of NaCl concentration.

In the sterile water treatment, the proline content in wheat seedlings was 24% greater at 10 mg mL^−1^ of NaCl concentration and was 25% greater at 20 mg mL^−1^, compared with 0 mg mL^−1^ of NaCl concentration (Table 3). Regardless of the salt level, the wheat seedlings treated with TL-6 strain increased the proline content significantly compared with the sterile water treatment. Averaged across the three (0, 10, 20 mg mL^−1^) NaCl levels, the wheat seedlings treated with TL-6 increased the proline content by 11% compared with sterile water treatment. 

### 2.4. Ascorbate and Glutathione Contents in Wheat Seedling

In the sterile water treatment, the ascorbate content of wheat seedlings was decreased by 22% at the 10 mg mL^−1^ of NaCl concentration and by 37% at the 20 mg mL^−1^ of NaCl stress, compared with 0 mg mL^−1^ of NaCl (Table 4). At each of the three NaCl levels, the wheat seedlings treated with TL-6 significantly increased the ascorbate content compared with sterile water treatment; the ascorbate content was increased by 6% (0 mg mL^−1^), 15% (10 mg mL^−1^) and 23% (20 mg mL^−1^), respectively. Similarly, a significant increase in glutathione content was detected in NaCl-stressed wheat seedlings treated with TL-6; especially the glutathione content of the TL-6 treated wheat seedlings was increased by 35% at the 10 mg mL^−1^ of NaCl stress compared with the seedlings in the sterile water treatment.

### 2.5. ROS Scavenging Enzymes Assay

**Figure 2:**
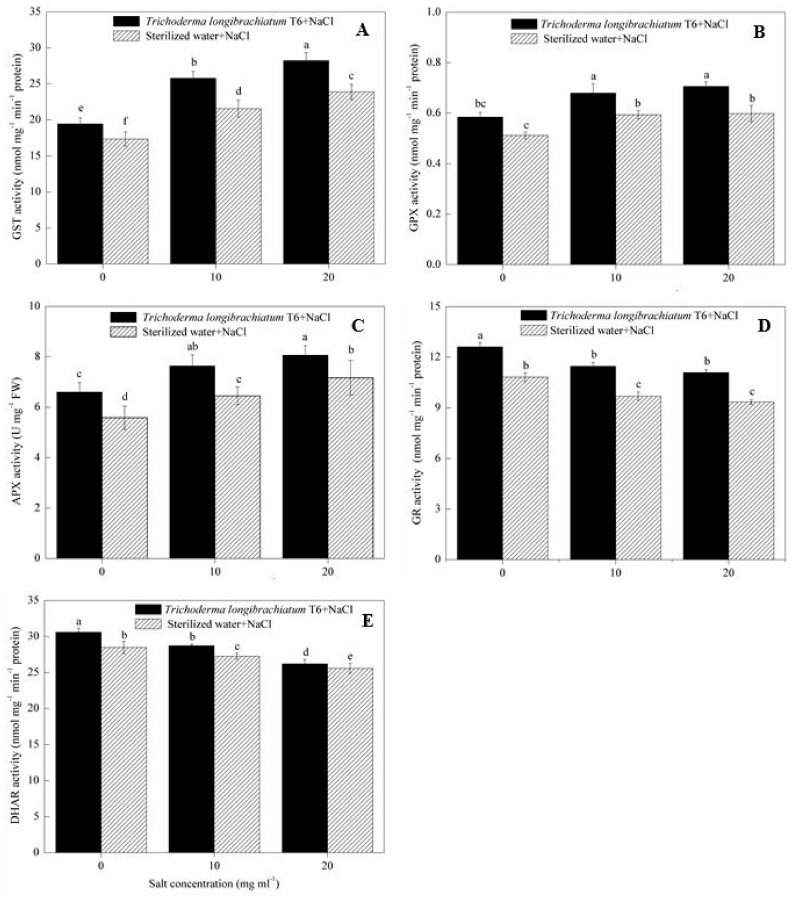
Effect of *Trichoderma longibrachiatum* T6 on the activity of (**A**) GST, (**B**) GPX, (**C**) APX, (**D**) GR and (**E**) DHAR in wheat seedling leaves under salt stress. The line bars represent the standard errors of the means. Different letters denote significant difference at the *p* < 0.05 level by Duncan’s new multiple range test (n = 12). The treatments are detailed in the footnote of Table 1.

In the sterile water treatment, the activity of GST, GPX and APX in wheat seedlings were increased after stressed with 10 and 20 mg mL^−1^ of NaCl solutions compared to the 0 mg mL^−1^ of NaCl-stressed plants (Figure 2), where GST activity was increased by 24% (10 mg mL^−1^) and 38% (20 mg mL^−1^) (Figure 2A), GPX activity was increased by 16% (10 mg mL^−1^) and 17% (20 mg mL^−1^) (Figure 2B), and APX activity was increased by 16% (10 mg mL^−1^) and 28% (20 mg mL^−1^) (Figure 2C), respectively. The activity of GST, GPX and APX were further increased in wheat seedlings after TL-6 was added to the NaCl treatment compared with sterile water treatment. At each NaCl level, wheat seedlings treated with TL-6 strain increased GST activity by 12%, 20% and 18%, respectively, at the NaCl concentration of 0, 10, and 20 mg mL^−1^ (Figure 2A); GPX activity was increased by 14%, 15% and 18%, respectively (Figure 2B); APX activity was increased by 18%, 19% and 13%, respectively (Figure 2C).

In contrast to the effect on the GST, GPX and APX, the activities of GR and DHAR in wheat seedlings were decreased with 10 and 20 mg mL^−1^ of NaCl solutions compared to the 0 mg mL^−1^ NaCl stress plants in sterile water treatment (Figure 2). The activity of GR was decreased by 10% (10 mg mL^−1^) and 14% (20 mg mL^−1^) (Figure 2D), and the activity of DHAR were decreased by 4% (10 mg mL^−1^) and 10% (20 mg mL^−1^) (Figure 2E), respectively, compared to the 0 mg mL^−1^ NaCl-stressed plants. However, compared with sterile water treatment, wheat seedlings treated with TL-6 strain increased the activity of GR by 17% (0 mg mL^−1^), 18% (10 mg mL^−1^) and 19% (20 mg mL^−1^) (Figure 2D), whereas the TL-6 treatment increased the activity of DHAR by 7%, 5% and 2% (Figure 2E), respectively.

### 2.6. ROS Scavenging Enzyme Gene Expression

**Figure 3:**
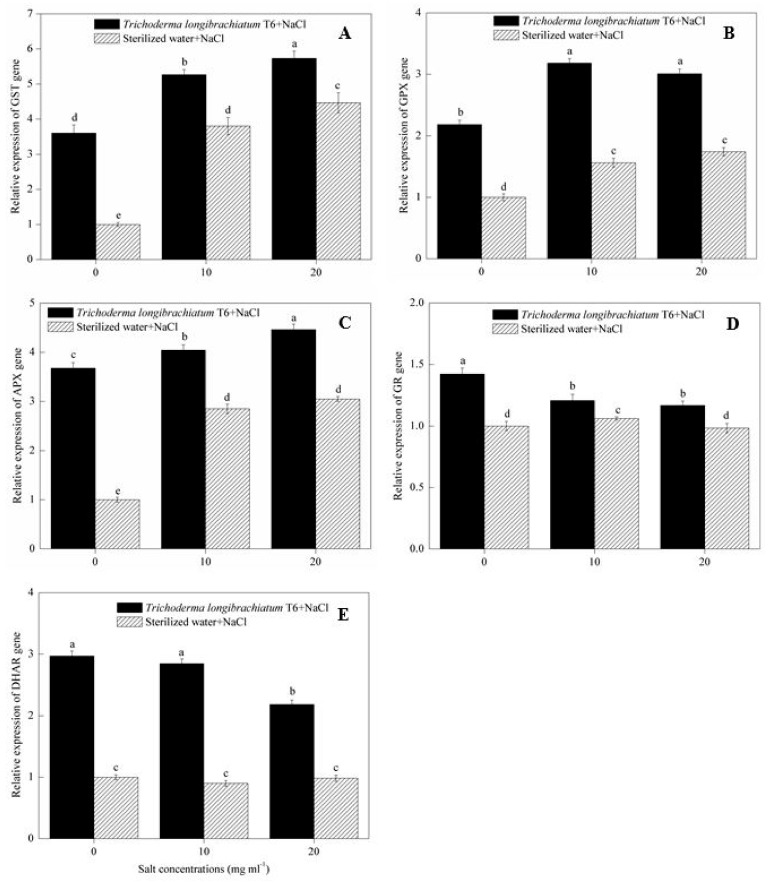
Effect of *Trichoderma longibrachiatum* T6 on the expression of (**A**) GST, (**B**) GPX, (**C**) APX, (**D**) GR and (**E**) DHAR genes in wheat seedling leaves under salt stress. The line bars represent the standard errors of the means. Different letters denote significant difference at the *p* < 0.05 level by Duncan’s new multiple range test (n = 12). The treatments are detailed in the footnote of Table 1.

In the sterile water treatment, NaCl stress (10 and 20 mg mL^−1^) induced higher transcript levels of the *GST* (Figure 3A), *GPX* (Figure 3B) and *APX* (Figure 3C) gene expression compared to the 0 mg mL^−1^ NaCl-stressed plants. At each of the three NaCl levels (0, 10, 20 mg mL^−1^), the application of TL-6 strain led to the upregulated expression of *GST* (Figure 3A), *GPX* (Figure 3B) and *APX* (Figure 3C) genes compared with sterile water treatment. However, compared to 0 mg mL^−1^ NaCl-stressed plants in sterile water treatment, NaCl stress (10 and 20 mg mL^−1^) induced the lower transcript levels of the *GR* (Figure 3D) and *DHAR* (Figure 3E) gene expression in sterile water treatment, but the transcript levels of the *GR* (Figure 3D) and *DHAR* (Figure 3E) gene expressions were upregulated significantly in seedlings treated with TL-6 under each of the three NaCl levels.

## 3. Discussion and Conclusions

Salt stress is an important abiotic factor limiting crop productivity as it reduces plant seeds germination, plant vigor and plant productivity, and affects different physiological and biochemical characteristics and mechanisms related to plant growth and development [18]. *Trichoderma* spp. are common and free-living fungi that are well researched for their growth promoting and biocontrol properties [19]. Although considerable amount of work has been published regarding the biocontrol properties of *Trichoderma*, not much attention has been paid in investigating the growth promoting and salt tolerance characteristics and mechanisms of *Trichoderma* spp. promoting wheat growth and enhancing plant tolerance to different levels of NaCl stress. Among several strategies applied to improve plant growth or tolerance to salt stress, our present study offers a new approach to alleviate salinity stress in wheat through seed treatment with salinity tolerant strain *Trichoderma longibrachiatum* T6 (TL-6). Our present study discovered that the salinity tolerant TL-6 strain effectively scavenged ROS through regulating the transcriptional levels of ROS scavenging enzyme gene expression to modulate the activity of ROS scavenging enzymes, and enhancing the nonenzymatic antioxidants in wheat seedling to activate the defense systems in wheat under different levels of salt stress. Our results will provide a vital theoretical basis and new insight for a more efficient application of TL-6 to activate the defense systems in wheat to adapt to saline conditions. 

In vitro and greenhouse experiments, different levels of NaCl stress decreased wheat seed germination and seedling growth significantly; this was expected. Several previous studies have demonstrated that NaCl stress is one of the most serious abiotic stresses affecting plant seeds germination, growth and biomass yield in many cereal and vegetable crops [20,21,22]. However, the supplementation of TL-6 alleviated the negative effects significantly in the present study. Similarly, the application of *Trichoderma* increased the biomass production of *Arabidopsis* [23], and the application of *T. harzianum* T22 enhanced tomato (*Lycopersicum esculentum* L.) seed germination under abiotic stresses [18]. 

In addition, studies have shown that salt stress can increase the production of ROS in plant cell that leads to plant membrane dysfunction and cell death [24]. To detoxify ROS, the plant can activate the antioxidant defense systems through inducing both enzymatic and nonenzymatic antioxidants. The enzymatic antioxidants mainly include superoxide dismutase, catalase, GPX, GST, and the enzymes of APX, DHAR, and GR. The latter three are the key enzymes involving in ascorbate–glutathione cycle and scavenging ROS [25]. The nonenzymatic antioxidants also play a role in scavenging ROS, including MDA, H_2_O_2_, proline, ascorbate and glutathione [25,26]. *Trichoderma* species can activate the antioxidant defense system to recycle the oxidized ascorbate and thus to enhance the plant tolerance to abiotic stresses in many plant species, such as *Arabidopsis*, tomato and cucumber [12,27]. In the present study, the presence of TL-6 strain significantly improved the activities of antioxidant enzymes, leading to the enhanced tolerance of wheat seedlings to salt stress. 

The present study also showed that *Trichoderma* species played a critical role in host plant metabolic processes in response to NaCl stress. The use of TL-6 strain increased proline content in wheat seedlings under NaCl stress, which helped maintain the cell osmo regulation [21] and energy storage [28]. Increased proline content enhanced the ability of plants to detoxify the accumulated ROS and protect the seedlings from oxidative damage [24,29]. In *Arabidopsis*, the treatment with the combination of salt and *Trichoderma* spp. increased proline content as compared to the treatment with salt alone [2]. Additionally, we found a decrease of MDA and H_2_O_2_ contents in wheat seedlings treated with TL-6 under salt stress. Similarly, the plants treated with beneficial arbuscular mycorrhizae fungi (AMF) have been reported to decrease the content of MDA in tomato [30], mulberry (*Morus alba* L.) [22] and okra (*Abelmoschus esculentus* L.) [31] while grown under salt stress. Rawat et al. (2013) [32] found that the content of H_2_O_2_ in chickpea (*Cicer arietinum* L.) was decreased with the inoculation of *T. harzianum*, compared with control plants. Furthermore, according to the published literature, application of exogenous salicylic acid significantly increased the contents of ascorbate and glutathione in wheat seedlings under salt stress [33], which in accordance with the results from our present study, the contents of ascorbate and glutathione were significantly increased after application of TL-6 under both saline and non-saline soil conditions. Meanwhile, our study found that with the increase level of salt stress, the content of ascorbate was significantly decreased, whereas the content of glutathione was significantly increased, which is in agreement with the previous reports [34,35].

Application of salicylic acid increased the transcripts of the genes encoding ascorbate and glutathione cycle enzymes [36,37], and the overexpression of these genes conferred an enhanced tolerance to salt or chilling stress [38,39]. In our study, the transcripts of the genes encoding ROS scavenging enzymes were increased significantly in wheat seedlings treated with the TL-6 strain under salinity and non-saline conditions. The degree of the expression of ROS scavenging enzyme genes was in coincidence with the corresponding activity of the ROS scavenging enzymes. Our results also indicate that the strain of TL-6 is able to regulate the transcriptional level of ROS scavenging enzyme genes effectively so as to enhance the enzymatic and nonenzymatic antioxidant defense systems in response to salt stress. 

Our present study offers a new approach to alleviate salinity stress in wheat through seed treatment with the salinity tolerant strain of TL-6. The beneficial role of TL-6 was reflected by stimulating seed germination, promoting seedling growth, and enhancing plant tolerance to saline stress. One novel possible mechanism is related to the enhanced roles of ROS scavenging enzymes and nonenzymatic antioxidants as signal to alleviate the negative effect of salt stress and activate the antioxidant defense systems in wheat seedlings response to salt stress. The present study is important for developing strategies that exploit these beneficial effects of the TL-6 on improving plant tolerance to abiotic stresses. 

Furthermore, several lines of study have shown that nitric oxide (NO) and ROS were putatively involved in a myriad of physiological events related to plant growth under salt, drought and other stress conditions [40,41,42]. Also, some previous studies revealed that ROS and NO play significant role in regulating of numerous physiological and pathological cell processes in mammals including regulation of immune responses, oncogenesis and neurodegeneration, as well as the relationship between ROS and NO under oxidative stress conditions [43,44]. However, the production of NO in wheat response to salt tress, and the effect of NO on the ROS accumulation in wheat after the application of TL-6 under salt stress that need to be addressed in the future studies.

## 4. Materials and Methods

Experiments were carried out at the Laboratory of Plant Pathology, College of Plant Protection, Gansu Agricultural University, and Gansu Provincial Biocontrol Engineering Laboratory of Crop Diseases and Pests. All treatments in the experiments described below had six replicates and each experiment was repeated twice over time, unless otherwise indicated.

### 4.1. Fungal Material 

*Trichoderma longibrachiatum* T6 (TL-6) was isolated from a rhizisphere saline-soil of a forest site nearby Tianshui, Gansu. The strain of TL-6 has since been collected at the China General Microbiological Culture Collection Center, in Beijing, with the patent number (CGMCC No.13183). The spore concentration of TL-6 was prepared according to the procedure described by Zhang et al. (2014) [45], with some modifications. The spore concentration in the suspension was determined with a hemacytometer, and the concentration was prepared and adjusted to 1 × 10^8^ spores mL^−1^. 

### 4.2. Seeds Treatment

The wheat (*Triticum aestivum* L.) cultivar ‘Yongliang 4’ provided by Gansu Academy of Agricultural Sciences (Lanzhou, China) was used in all the experiments. The cultivar has been well adapted at the major wheat growing areas of northwest China. Wheat seeds with a uniform size were surface-sterilized with 1% (v/v) NaOCl for 5 min and then with 95% (v/v) ethanol for 5 additional minutes. After disinfection, all the seeds were rinsed with sterile water, and then were soaked in TL-6 spore suspension at the concentration of 1 × 10^8^ spores mL^−1^ for 12 h. The control seeds were soaked in sterile water for 12 h.

### 4.3. Effect of TL-6 on Wheat Seed Germination under NaCl Stress

Wheat seeds (50 seeds) treated with the spore suspension of TL-6 or sterile water were germinated in 9-cm diameter Petri dishes that contained 10 mL of NaCl solutions, and the dishes were covered with a layer of absorbent cotton and blotter papers. The germination was tested at the NaCl concentrations of 0, 10 and 20 mg mL^−1^. Petri dishes were incubated at 25 ± 1 °C with supplemental day/night lighting of 16/8 h. Seed germination was recorded at 24 h intervals for a further period of 5 days. Seed germination potential was determined at Day 3 after incubation, whereas seed germination rates and germination index were determined at Day 5. The average length of plumule and radicle were determined at Day 7.

Seeds germination rate (SGR), germination potential (GP) and germination index (GI) were calculated according to the formula described by Niu et al. (2013) [46].

SGR (%) = (NSG/TNS) × 100(1)

where NSG is number of seeds germinated after 5 days of incubation, and TNS is total number of seeds in each Petri dish.

GP (%) = (NSG/TNS) × 100(2)

where NSG is number of seeds germinated after 3 days of incubation, and TNS is total number of seeds in each Petri dish.

GI (%) = ∑NGSi/Ti × 100(3)

where NGSi is number of germinated seeds in a given time, and Ti is the incubation time (day).

### 4.4. Effect of TL-6 on Wheat Seedling Growth under NaCl Stress in Greenhouse

Experiments were carried out in a greenhouse with constant temperature of 25°C±0.5, supplemental day/night lighting of 16/8 h, and relative humidity of 65%. Wheat seeds (80 seeds) with a uniform size were planted in 10-cm diameter pots that contained 300 g of sterilized soil. A total of 50 seedlings per pot were kept through thinning at Day 12 after emergence. The experiments included two group treatments: (i) wheat seeds were soaked with the spore suspension of TL-6 and inoculated at 0, 10 and 20 mg mL^−1^ of NaCl concentrations, and (ii) wheat seeds were soaked with sterile water and inoculated at 0, 10 and 20 mg mL^−1^ of NaCl concentrations. Each of the NaCl-treated pots was irrigated with 25 mL of NaCl solution whereas the 0 mg mL^−1^ of NaCl concentration treatment was irrigated with 25 mL of sterile water. Plants were grown in a greenhouse (25°C) with supplemental day/night lighting of 16/8 h. The experiment was arranged using a completely randomized design, and each pot was irrigated with 200 mL of sterile distilled water at regular intervals. The seedling height, root length, shoot and root fresh and dry weight, and the biochemical, physiological and molecular parameters were determined at Day 35. 

### 4.5. Lipid Peroxides and Proline Contents Determination

Lipid peroxides content in plants is commonly considered as an indicator of oxidative damage caused by abiotic stress. At the present study, the accumulation of MDA in wheat seedlings treated with TL-6 strain or sterile water under the different concentrations of NaCl solutions (0, 10, and 20 mg mL^−1^) was measured using the method of Demiral and Turkan (2005) [47] and Tian et al. (2015) [48], with minor modifications. For the determination of the accumulation of MDA in wheat seedlings, 0.5 g of leave samples were grounded in 2.5 mL of 0.1% trichloroacetic acid (TCA) and the homogenate was centrifuged for 10 min at 10,000× *g*. An aliquot of 1 mL of the supernatant was mixed with 4 mL of 20% TCA containing 0.5% thiobarbituric acid at 90°C for 30 min, and then cooled quickly on an ice bath. Afterwards, the reaction mixture was centrifuged at 10,000× *g* for 15 min and the absorbance of supernatant was recorded at 532 nm wavelength. Measurements were corrected for unspecific turbidity by subtracting the absorbance at 600 nm. The content of MDA was expressed as µmol g^−1^ FW. 

The content of proline in wheat seedlings was determined by following the procedure described by Bates et al. (1973) [49]. In brief, 0.5 g of wheat leaves were homogenized in 10 mL of 3% aqueous sulphosalicylic acid, and the homogenate was centrifuged at 10,000× *g* for 10 min, and then filtered through Whatman Paper No.3 filter for three times. After that, 1 mL of supernatant was mixed with 1 mL of glacial acetic acid and acid ninhydrin at 100°C for 1 ho and then the reaction was quickly stopped in an ice-bath. Afterwards, the reaction mixture was extracted with 2 mL of toluene, and mixed with a tube stirrer for 30 s. The chromophore containing toluene was aspirated from the aqueous phase at room temperature, and the absorbance was measured at 520 nm wavelength on spectrophotometer and the toluene was used as blank.

The proline concentration (µmol per g of FW material) was determined from a standard curve and calculated by following the method of Bates et al. (1973) [49], as follow:

Proline concentration = [(µg proline per mL × mL toluene)/115.5 µg per µmol]/[(g sample)/5](4)

### 4.6. H_2_O_2_ Assay in Wheat Seedling

The content of H_2_O_2_ in wheat seedlings was determined following the method of Jena and Choudhuri (1981) [50] with some modifications. Seedling leaves of 0.5 g were homogenized with 3 mL of K-phosphate buffer (50 mM, pH 6.5). The extraction was centrifuged at 10,000× *g* for 20 min at 4°C, and then 1 mL of 0.1% titanium tetrachloride with 20% sulfuric acid (v/v) was added to 3 mL of extraction supernatant. The reaction mixture was centrifuged at 10,000× *g* for 10 min, and the content of H_2_O_2_ was calculated by measuring the absorption of supernatant at 410 nm wavelength, and expressed as µmol g^−1^ of FW.

### 4.7. Determination of Ascorbate and Glutathione

The crude extraction for the determination of ascorbate and glutathione contents in wheat seedlings was extracted by following the procedure of Foyer et al. (1983) [51], Hasanuzzaman and Fujita (2011) [35], with some modifications. The frozen wheat seedling leaves of 1.0 g were homogenized in 3 mL of 5% ice-cold metaphosphoric acid buffer (pH 7.0) that contained 1 mM ethylenediaminetetraacetic acid. The crude extraction was centrifuged at 10,000× *g* for 20 min, and the supernatant was used for the determination of ascorbate and glutathione contents. 

Ascorbate content was determined following the method of Huang et al. (2005) [34] with some modifications. The supernatant of crude extraction (1 ml) was neutralized with 0.5 M K-phosphate buffer (pH 7.0) at room temperature. The ascorbate content was assayed spectrophotometrically at 265 nm in 100 mM K-phosphate buffer (pH 7.0) with 0.5 unit of ascorbate oxidase. A specific standard curve with ascorbate was used for quantification and calculation the content of ascorbate. 

An aliquot of supernatant (1 ml) was neutralized with 0.5 M K-phosphate buffer (pH 7.0) at room temperature. Reduced glutathione is oxidized by 5, 5′-dithio-bis (2-nitrobenzoic acid) and reduced by nicotinamide adenine dinucleotide 2′-phosphate reduced tetrasodium salt hydrate (NADPH) in the presence of GR. The reaction mixture was used for the determination of the content of glutathione and evaluated by the rate of absorption changes at 412 nm of 2-nitro-5-thiobenzoic acid generated from the reduction of 5, 5′-dithio-bis (2-nitrobenzoic acid). A specific standard curve with known concentrations of reduced glutathione and oxidised glutathione were used for quantification. The content of glutathione was calculated by subtracting oxidised glutathione from total reduced glutathione [52].

### 4.8. ROS Scavenging Enzymes Extraction and Determination 

Wheat seedling leaves of 1.0 g were homogenized in 5 mL of 100 mM Tris-HCl (pH 7.5) that contained 5 mM dithiothreitol, 1 mM ethylenediaminetetraacetic acid, 10 mM magnesium chloride, 5 mM magnesium acetate, 1.5% Polyvinylpyrrolidone, 1 mM Phenylmethanesulfonyl fluoride and 1 μg mL^−1^ aprotinin. The sample filtration was centrifuged at 12,000 rpm for 10 min, and the supernatant was collected for the determination of the activity of ROS scavenging enzymes. 

APX activity was determined according to the method as described by Nakano and Asada (1981) [53], and expressed as U mg^−1^ FW of protein. The enzyme extract solution in a final volume of 1 mL that contained 50 mM K-phosphate buffer (pH 7.0), 0.5 mM ascorbate, 0.1 mM H_2_O_2_ and 0.1 mM ethylenediaminetetraacetic acid. The activity was measured by observing the decrease of reaction mixtures in absorbance at 290 nm.

GST activity was assessed following the procedure of Hasanuzzaman and Fujita (2013) [52] with some modifications. A final volume of 1 mL enzyme solution that contained 100 mM Tris-HCl buffer (pH 6.5), 1 mM 1-chloro-2, 4-dinitrobenzene and 1.5 mM reduced glutathione. The increase in absorbance was measured at 340 nm, and GST activity was expressed as nmol mg^−1^ protein min^−1^.

GPX activity was measured spectrophotometrically with the method of He et al. (2006) [54] and Elia et al. (2003) [55] using H_2_O_2_ as a substrate. The enzyme solution (1 ml) that consisted of 50 mM K-phosphate buffer (pH 7.0), 0.12 mM NADPH, 1 mM ethylenediaminetetraacetic acid, 2 mM reduced glutathione, 1 mM NaN_3_, 1 unit GR and 0.6 mM H_2_O_2_. The absorbance of reaction mixture was assayed at 340 nm wavelength and GPX activity was expressed as 0.1 nmol oxidized NADPH mg^−1^ protein min^−1^.

DHAR activity was determined according to Nakano and Asada (1981) [53] by assaying the decrease in absorbance of dehydroascorbate at 265 nm. The enzyme solution (1 ml) that contained 50 mM K-phosphate buffer (pH 7.0), 0.1 mM dehydroascorbate, and 2.5 mM reduced glutathione. DHAR activity was expressed as nmol mg^−1^ protein min^−1^.

GR activity was measured according to the method of Foyer and Halliwell (1976) [56]. The supernatant was contained 1 mL of enzyme solution (1 μmol ethylenediaminetetraacetic acid, 3.3 mg oxidised glutathione, 0.1 mg NADPH and 87 μmol Tris buffer). Corrections were made for NADPH oxidation in the absence of oxidised glutathione. The absorbance of reaction mixture was assayed at 340 nm wavelength and GR activity was expressed as 0.1 nmol oxidized NADPH mg^−1^ protein min^−1^ [57]. 

### 4.9. Total RNA Extraction and First Strand cDNA Synthesis 

Wheat seedlings of 35 days old (200 mg sample) in all treatments including those treated with TL-6 strain or sterile water under different concentrations of NaCl solutions (0, 10 and 20 mg mL^−1^) were used to extract the total RNA. The total RNA was conducted by following the manufacturer′s instruction of Tiangen RNA Simple Total RNA Kit (Tiangen Biotechnology, Beijing, China). After that, one microgram of total RNA was reversely transcribed using the SYBR Prime Script RT-PCR kit II (Takara Biotechnology, Dalian, China) for the first-strand cDNA synthesis with oligo-dT_18_ primer priming method according to the manufacturer’s recommendations. 

### 4.10. Quantitative Real-Time PCR (qRT-PCR) Analysis

The expression level of genes encoding ROS scavenging enzymes was determined in wheat seedlings after treated with TL-6 strain or sterile water under different concentrations of NaCl solutions. qRT-PCR was performed using a SYBR Premix Ex Taq kit (Takara Biotechnology, Dalian, China) following the manufacturer′s instruction. The sequences of the forward and reverse primer pairs (*GPX*, *GST*, *APX*, *DHAR*, *GR* and *Actin* genes) used for qRT-PCR analysis were designed according to the EST (Expressed sequence tag) sequences of wheat in NCBI (National Center for Biotechnology Information) [33,58] using Primer Express 5.0 software that amplifies the target genes (Table 5). The actin gene of wheat was used as an internal control. The relative expression of *GPX*, *GST*, *APX*, *DHAR* and *GR* genes was determined using the method of 2^−ΔΔCt^ [59].

### 4.11. Statistical Analysis

The data were tested in each experiment included TL-6 or sterile water treatments at the different concentrations of salt stress. Each experiment had six replications and was repeated twice over time, but there were no significant interactions between the two runs and treatments, and thus the results obtained from the two runs of experiments are presented as average (mean) with the standard error. Data were analyzed using two-way ANOVA in SPSS Version 16.0 (SPSS Inc., Chicago, IL, USA), and mean comparisons were made using Duncan’s new multiple range test and the significance was considered at *P*< 0.05.

## Figures and Tables

**Table 1:** Effect of the strain *Trichoderma longibrachiatum* T6 on wheat seed germination at the different NaCl solutions.

Treatment	Salt Concentrations (mg mL^−1^)	Germination Rate (%)	Germination Potential (%)	Germination Index (%)
*Trichoderma longibrachiatum* T6	0	96.0 ± 1.2^a^	89.0 ± 1.2^a^	74.2 ± 0.7^a^
	10	83.3 ± 1.4^b^	65.0 ± 1.2^c^	52.2 ± 1.2^c^
	20	68.3 ± 1.7^d^	51.3 ± 1.4^e^	40.2 ± 0.7^e^
Sterile water	0	93.3 ± 1.2^a^	82.0 ± 1.2^b^	68.1 ± 1.2^b^
	10	78.0 ± 2.3^c^	61.3 ± 0.8^d^	44.9 ± 1.2^d^
	20	63.3 ± 1.2^e^	47.0 ± 1.2^f^	38.0 ± 1.7^e^

Data are means ± standard error of replicates, and the germination rate, potential and index were determined 5, 3 and 5 days after treatment, respectively. Different letters in the same column denote significant differences at the *p* < 0.05 level by Duncan’s new multiple range test (n = 12). In the three TL-6 treatments, wheat seeds were presoaked with the suspension of *Trichoderma longibrachiatum* (TL-6) spores for 12 h, whereas in the three sterile water treatments, wheat seeds were presoaked with sterile water only.

**Table 2:** Effect of the strain *Trichoderma longibrachiatum* T6 on wheat seedling growth at the different NaCl solutions.

Treatment	Salt Concentration (mg mL^−1^)	Shoot Length (cm plant^−1^)	Root Length (cm plant^−1^)	Fresh Weight (g plant^−^^1^)	Dry Weight (g plant^−^^1^)
*Trichoderma longibrachiatum* T6	0	31.24 ± 0.7^a^	24.79 ± 0.6^a^	0.47 ± 0.01^a^	0.14 ± 0.02^a^
	10	31.05 ± 0.7^a^	22.55 ± 0.5^b^	0.43 ± 0.01^ab^	0.11 ± 0.01^abc^
	20	28.67 ± 0.5^b^	21.58 ± 0.4^c^	0.34 ± 0.02^c^	0.09 ± 0.01^bc^
Sterile water	0	28.55 ± 0.6^b^	22.24 ± 0.3^b^	0.41 ± 0.02^b^	0.12 ± 0.01^ab^
	10	27.43 ± 0.6^bc^	20.21 ± 0.5^c^	0.35 ± 0.02^c^	0.09 ± 0.02^bc^
	20	24.22 ± 0.4^c^	18.65 ± 0.4^d^	0.29 ± 0.02^d^	0.07 ± 0.01^c^

Data are mean ± standard error of replicates, and the shoot and root length, fresh and dry weights of wheat seedling were determined 35 days after treatment. Different letters in the same column denote significant differences at the *P*<0.05 level by Duncan’s new multiple range test (n = 12). The treatments are detailed in the footnote of Table 1.

**Table 3:** Effect of *Trichoderma longibrachiatum* T6 on MDA, H_2_O_2_ and proline contents in wheat seedlings under salt stress.

Treatment	Salt Concentration (mg mL^−1^)	MDA Content (µmol g^−1^ FW)	H_2_O_2_ Content (µmol g^−1^ FW)	Proline Content (µmol g^−1^ FW)
*Trichoderma longibrachiatum* T6	0	9.29 ± 0.6^d^	5.84 ± 0.24^d^	12.28 ± 1.2^c^
	10	11.46 ± 1.0^c^	6.57 ± 0.26^cd^	15.58 ± 0.9^a^
	20	12.35 ± 0.8^b^	6.75 ± 0.27^bc^	15.46 ± 0.7^a^
Sterile water	0	10.88 ± 0.9^c^	6.50 ± 0.27^cd^	11.21 ± 1.1^d^
	10	14.78 ± 1.0^ab^	7.43 ± 0.27^ab^	13.93 ± 1.0^c^
	20	15.33 ± 0.7^a^	8.07 ± 0.12^a^	14.01 ± 0.9^b^

Data are means ± standard error of replicates and those in a column followed by different letters are significantly different at *p* < 0.05, based on Duncan’s new multiple range test using two-way ANOVA (n = 12). The treatments are detailed in the footnote of Table 1. FW represents fresh weight.

**Table 4:** Effect of *Trichoderma longibrachiatum* T6 on the contents of glutathione and ascorbate in wheat seedling leaves under salt stress.

Treatment	Salt Concentration (mg mL^−1^)	Glutathione (µmol g^−1^ FW)	Ascorbate (µmol g^−1^ FW)
*Trichodermalongibrachiatum* T6	0	0.99 ± 0.05^cd^	0.88 ± 0.02^a^
	10	1.24 ± 0.04^b^	0.75 ± 0.02^b^
	20	1.46 ± 0.05^a^	0.64 ± 0.02^c^
Sterile water	0	0.85 ± 0.01^e^	0.83 ± 0.01^a^
	10	0.92 ± 0.04^de^	0.65 ± 0.02^c^
	20	1.11 ± 0.05^c^	0.52 ± 0.03^d^

Data are means ± standard error of replicates and those in a column followed by different letters are significantly different at *p* < 0.05, based on Duncan’s new multiple range test using two-way ANOVA (n = 12). The treatments are detailed in the footnote of Table 1.

**Table 5:** DNA sequences of qRT-PCR primers for the determination of the ROS scavenging enzyme gene expression in wheat seedlings.

Genes	Premiers Sequence (5′-3′)
*APX*	F: AAAACCACCTACTGCCACCCTATC
	R: AGCATTCGCTCCATGACTCAACT
*GST*	F: AGCTCTTGGCGTCTTGGCT
	R: AGGCTTCCCCTTGGAGCAC
*GPX*	F: CCTAACTAACTCCAACTACACC
	R: TCCTGCCCACCAAACTGAT
*GR*	F: ATGAATACTCCCGTACATCAGT
	R: TTTGTTACATCACCCACAGC
*DHAR*	F: AGAAGTTTACGCCCTTCGGC
	R: ACAAGTGATGGAGTTGGGT
*Actin*	F: CCGTGGTGATGTTGTGCCAAAGGA
	R: CGACGACACTGGTGGAGTTGGAGA

Note: F represents forward, R represents reverse.
